# Analysis of composition-based metagenomic classification

**DOI:** 10.1186/1471-2164-13-S5-S1

**Published:** 2012-10-19

**Authors:** Susan Higashi, André da Motta Salles Barreto, Maurício Egidio Cantão, Ana Tereza Ribeiro de Vasconcelos

**Affiliations:** 1 Laboratório Nacional de Computação Científica (LNCC), Petrópolis, RJ, Brazil; 2 Embrapa Suínos e Aves, Concórdia, SC, Brazil

**Keywords:** Metagenomics, classification problem, taxonomic classification

## Abstract

**Background:**

An essential step of a metagenomic study is the taxonomic classification, that is, the identification of the taxonomic lineage of the organisms in a given sample. The taxonomic classification process involves a series of decisions. Currently, in the context of metagenomics, such decisions are usually based on empirical studies that consider one specific type of classifier. In this study we propose a general framework for analyzing the impact that several decisions can have on the *classification problem*. Instead of focusing on any specific classifier, we define a generic score function that provides a measure of the difficulty of the classification task. Using this framework, we analyze the impact of the following parameters on the taxonomic classification problem: (i) the length of *n*-mers used to encode the metagenomic sequences, (ii) the similarity measure used to compare sequences, and (iii) the type of taxonomic classification, which can be conventional or hierarchical, depending on whether the classification process occurs in a single shot or in several steps according to the taxonomic tree.

**Results:**

We defined a score function that measures the degree of separability of the taxonomic classes under a given configuration induced by the parameters above. We conducted an extensive computational experiment and found out that reasonable values for the parameters of interest could be (i) intermediate values of n, the length of the *n*-mers; (ii) any similarity measure, because all of them resulted in similar scores; and (iii) the hierarchical strategy, which performed better in all of the cases.

**Conclusions:**

As expected, short *n*-mers generate lower configuration scores because they give rise to frequency vectors that represent distinct sequences in a similar way. On the other hand, large values for n result in sparse frequency vectors that represent differently metagenomic fragments that are in fact similar, also leading to low configuration scores. Regarding the similarity measure, in contrast to our expectations, the variation of the measures did not change the configuration scores significantly. Finally, the hierarchical strategy was more effective than the conventional strategy, which suggests that, instead of using a single classifier, one should adopt multiple classifiers organized as a hierarchy.

## Background

Rather than considering a single species in pure culture, metagenomics goes beyond and focuses on the exploration of entire microbial communities [1]. This focus is possible only because of the recent improvements in sequencing technology. As is typical of new concepts, the emergence of this new paradigm has brought up some new challenges. Among them, the manipulation and analysis of short reads deserves special attention.

In some cases, the phylogenetic diversity of a microbial community is not well covered and, as a consequence, only a few reads can be assembled [2]. Hence, one of the first steps of a large-scale metagenomic analysis is to estimate the phylogenetic distribution of the sample. One approach to perform this task is the taxonomic classification of the reads, which is the assignment of these reads into phylogenetic categories [3].

Essentially, there are three approaches to classifying sequences into taxonomic categories. One possibility is to focus on conserved gene markers (such as rRNA 16S) to identify the source organism of the read. Because rRNA is well conserved, this approach produces an accurate taxonomic classification of the reads. Nevertheless, because only a small fraction of the sequences contain these gene markers, most of the reads of a metagenomic sample cannot be classified using this approach [4].

Taxonomic classification can also be based on sequence similarity, that is, the alignment of metagenomic reads to a reference dataset (for example using BLAST [5]). This approach is an accurate method, as long as a similar sequence is present in the database--which is not always true for metagenomic projects [3]. Some examples of off-the-shelf software for metagenomic analysis based on sequence similarity are CARMA [6] and Megan [7].

Yet another way to perform the taxonomic classification is to rely on a set of features that is induced by the sequences of nucleotides, producing the so-called *composition-based classification *[8]. Some features employed in this case are: codon usage, GC content, and oligonucleotide frequency (henceforth *n*-mer frequency). The latter is usually considered to be a good choice, because the *n*-mer frequencies carry phylogenetic signals that are useful for extracting common patterns between organisms at different taxonomic levels [9-11]. The following are some examples of software for taxonomic classification based on *n*-mer frequencies: Phylopythia [4] implements a support vector machine for classifying sequences that are larger than 3 kbp, Phymm [12] uses interpolated Markov modes (IMM) to classify reads with at least 100 bp, TACOA [8] merges the k-nearest-neighbor (k-NN) algorithm with kernelized learning strategies to handle sequences from 800 bp to 50 kbp, and Treephyler [3] uses hidden Markov models (HMM) to classify reads of 200 bp.

This work focuses on composition-based classification using *n*-mer frequencies to encode genomic sequences. Such an approach involves a series of decisions, regardless of the specific classifier chosen to perform the task. Usually, these decisions are based on a set of preliminary experiments that account for one particular type of classifier [4,13]. These studies provide valuable information regarding the performance of a given category of classifier; however, because they are biased by the peculiarities of the classifier of choice, they provide little insight about the characteristics of the classification problem itself. This paper presents a general framework for the empirical assessment of the impact that several decisions have on the degree of separability of taxonomic classes. Thus, instead of focusing on any classifier in particular, we focus our study on the classification problem.

Here we refer to a specific configuration of the classification problem as the setting induced by the following three features: (i) the length of the *n*-mer word used to encode the DNA sequences; (ii) the similarity measure adopted to compare the sequences; and (iii) the strategy used to assign sequences to taxonomic classes, which can be the conventional approach, in which the sequences are considered independently, or the hierarchical approach, in which the taxonomic context of each DNA fragment is accounted for. The goal of the current work is to serve as a guideline for the development of composition-based metagenomic classifiers by providing some intuition as to how the difficulty of the taxonomic classification problem changes with respect to the variation in the features described above.

## Methods

### Acquisition of datasets

We used two types of data: (i) complete genomes; and (ii) synthetic metagenomic fragments. These datasets are described in the following sections.

#### Complete genomes

The genomes were obtained from GenBank, the NCBI database of genetic sequences [14]. We used only microbial sequences, because the majority of metagenomic studies are focused on this type of organism [15]. We considered all 1, 032 microbial genomes sequenced until January, 2010. Among these, 497 sequences had to be removed because they had incomplete taxonomic lineage or undefined nucleotides. Hence, the actual number of genomes used was 535, which encompassed the domains Bacteria and Archaea.

#### Synthetic metagenomic fragments

The synthetic fragments were generated by the program MetaSim [16] using the genomes described above. MetaSim is a metagenomic sequence simulator that can be used to create sets of synthetic fragments reflecting the taxonomic composition of typical metagenomic scenarios. A total of 23, 000 fragments with ~ 400*bp *was generated under the sequencing conditions of Roche's 454 pyrosequencer [17].

### Preprocessing of datasets

We now describe how we preprocessed the data to perform our analysis.

#### Calculating n-mer frequencies

**Figure 1:**
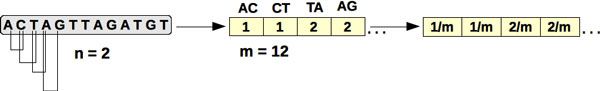
**Process of counting n-mer frequencies**. Given a value for *n*, the first step is generating all of the *n*-mer words that are possible. In the next step, we count the number of times that each word appears in the sequence. Finally, we normalize the frequency vector by dividing each number of occurrences by the total number of *n*-mers.

To encode the nucleotide sequences we calculated the *n*-mer frequencies in each (meta)genomic sequence. To do so, we counted the number of occurrences of all possible *n*-mers in a given sequence, considering an overlap of *n - *1 nucleotides (that is, we started from position 1 to n, then from position 2 to n+1, and so on). This strategy gives rise to a 4*^n^*-dimensional vector whose elements represent the number of occurrence of each possible *n*-mer. We then divided the elements of such a vector by the total number of *n*-mers contained in the sequence. For the experiments with Kullback-Leibler (KL) divergence we used a slightly different approach to count the *n*-mers, because the strategy above could lead to a division by zero (see Equation 4). In particular, we assumed that each *n*-mer had occurred at least once, a method usually referred to in the literature as "add-one smoothing" [18]. In the end, each sequence is represented by a vector of *n*-mer frequencies (hereafter, "vector of frequencies"). We will sometimes refer to a vector of frequencies as simply a "sequence" when there is no risk of misinterpretation. Figure 1 illustrates the process described above.

#### Determining taxonomic lineage

To associate the sequence with its corresponding taxonomic lineage we used the information available at *NCBI Taxonomy *and BioPerl, a toolkit for the manipulation of genomic data [19]. The result of this process was a vector comprising seven positions that were filled out with NCBI *taxids *(taxonomy identifiers) corresponding to each one of the seven taxa: domain, phylum, class, order, family, genus, and species.

### Score functions

The next step is implementing a score function, which provides, under a specific configuration, a score for the degree of separability of the taxonomic classes. To formally define this function, we will adopt the following notation. *D *= {*G*, *F*} is the dataset, in which *G *represents the genomic sequences and *F *is the metagenomic synthetic fragments. *T *= {*do*, *ph*, *cl*, *or*, *fa*, *ge*, *sp*} is the taxon set, which represents the sequence's taxonomic lineage. *N *= {1, 2, . . . , 10} is the set of lengths of *n*-mers and *S *= {1, 2, ∞, *kl*} represents the set of similarity measures, where 1 is the 1-norm distance (Equation 1), 2 represents the 2-norm (Euclidean) distance (Equation 2), and ∞ is the ∞-norm distance (Equation 3); *kl *is the Kullback-Leibler divergence (Equation 4).

(1) $$s_{1}\left(x,y\right)=\overset{4^{n}}{\underset{i=1}{\sum }}|x_{i}-y_{i}|,$$

(2) $$s_{2}\left(x,y\right)={\left(\overset{4^{n}}{\underset{i=1}{\sum }}|x_{i}-y_{i}{|}^{2}\right)}^{1/2},$$

(3) $$s_{\infty }\left(x,y\right)=\underset{i=1,2,...,4^{n}}{\text{max}}|x_{i}-y_{i}|,$$

(4) $$s_{\text{k}l}\left(x,y\right)=\overset{4^{n}}{\underset{i=1}{\sum }}x_{i}\text{ln}\left(\frac{x_{i}}{y_{i}}\right).$$

*A *={*c*, *h*} is the set of score measures. The element *c *represents the conventional score measure, in which the configuration is scored considering the sequence separately, and the element *h *is the hierarchical score measure, in which the configuration is scored with respect to the sequence's taxonomic context (see below). Considering this notation the score function is defined as follows:

(5) f:D×T×N×S×A→[0,1].

Thus, *f *(*d*, *t*, *n*, *s*, *a*) = *y *represents a score *y *to the dataset *d*, considering the taxon *t*, using a *n*-mer length of *n *to encode the sequences and the similarity measure *s *to check how similar the sequences are and, finally, using the score measure *a*. In other words, the score *y *is a measure of the degree of separability of the taxonomic classes in *d *at level *t *under the specific configuration induced by *n*, *s*, and *a*.

We now describe how we defined the score measures that were used to evaluate the classification problem.

#### Conventional score measure

We want to assess the "separability" of the taxonomic classes under a given configuration. A straightforward way to do so would be to choose a specific type of classifier and then measure its classification accuracy for each possible combination of values for (*d*, *t*, *n*, *s*, *a*) (using cross-validation, for example [20]). Note that in this case we would be measuring the difficulty of the problem under the assumptions made by that specific classifier. For example, if we adopted a linear model such as the Naive Bayes classifier, then we would be measuring how well classes can be separated by a hyperplane [20]. Therefore, if we want to make no assumptions regarding the "shape" of the classes, the correct approach would be to use a nonlinear model capable of representing any boundary between the classes (such as a support vector machine using an appropriate kernel [21]). However, such an approach would require an expensive cross-validation process to determine the correct level of complexity of the model under each configuration (using, for example, regularization [20]).

We want a measure of the separability of the classes that can be efficiently computed and at the same time makes no strong assumptions regarding the shapes of the classes. A possible way of solving this problem is to base our measure on this simple observation: given a set of objects that belong to different classes, the level of separability of the classes can be assessed by the fraction of objects whose closest neighbor belongs to the same class. Note that, under this criterion, if the boundaries between the classes are well defined, then the set of objects will usually be considered to be separable, regardless of the shape of the classes. Therefore, this simple measure is an efficient way of assessing the degree of overlap between classes.

Algorithm 1 presents a detailed description of the computation of the proposed separability measure. Given a configuration (*d*, *t*, *n*, *s*), for each sequence in *d*, we calculate its nearest neighbor (NN) and check whether both sequences belong to the same class at the taxonomic level *t*. If so, then we add 1 to the configuration score. The result is then normalized to fall in the interval [0,1]. We call this approach the *conventional score *measure.

**Algorithm 1**: conventional_score(*d*, *t*, *n*, *s*)

/* Computes the conventional score for a given set of DNA sequences */

**Input**: *d *∈ *D*, *t *∈ *T*, *n *∈ *N*, *s *∈ *S*

**Output**: Conventional score

1 score ← 0

2*m *← 0

3**foreach ***sequence d_i _*∈ *d ***do**

4 **if ***d_i _is not the only representative of its class in d at level t ***then**

5 *m *← *m *+ 1

6 *d_j _*← *NN*(*d_i_*, *d*, *n*, *s*) ;/* nearest neighbor of *d_i _*in *d *using *n*-mers and measure s */

7 **if **class(*d_i_*) = class(*d_j_*) at taxonomic level *t ***then **score ← score + 1

8 **return **score/*m*

Note that, if a genome is the only representative of its taxonomic group, then its nearest neighbor will necessarily belong to another class, which biases downwards the score measure shown in Algorithm 1. For this reason, we classify a genome only if it is not the unique example of its taxonomic class (line 4 of Algorithm 1). In the dataset used in our experiments, classes with a single member occur only at the taxonomic level of species. Specifically, out of 535 genomes used in the experiments, 328 were the unique representatives of their species.

As shown in Algorithm 1, the conventional score is the percentage of sequences that have the same lineage as their nearest-neighbors at a given taxonomic level. Incidentally, this approach is similar to using the *k*-Nearest-Neighbor (*k*-NN) classifier with *k *= 1 (except that in the latter case we would not eliminate classes with a single representative) [22]. This approach is in accordance with our objective of focusing our analysis on the classification problem, because the 1-NN classifier does not make strong assumptions regarding the shape of the classes [20].

#### Hierarchical score measure

Given the hierarchical structure of the taxonomic classification task, one might wonder whether it is a good strategy to decompose the problem into simpler sub-problems that are defined at each taxonomic level. More specifically, instead of using a single classifier, one would have a hierarchy of classifiers that are organized according to the taxonomic tree. In this case, a given DNA sequence would be classified as follows: first, a classifier at the highest hierarchical level would determine the domain to which the sequence belongs. Then, the DNA sequence would be classified at the next hierarchical level, the phylum, with the particular classifier used to do so determined by the domain the sequence was assigned to at one level above. Following the same reasoning, the sequence would then be passed on to the classifier that is responsible for the specific phylum that it was assigned to, and so on, until the desired taxonomic level had been reached. This classification strategy has been used before in the literature [4,23].

Note that, to compare the hierarchical scoring measure with the conventional measure, we cannot simply apply Algorithm 1 at each taxonomic level, because the nearest neighbor of a given sequence defines its classification at all of the taxonomic levels (and thus the hierarchical score would coincide with the conventional score). Since we do not want to introduce any bias in our analysis, we must define a score measure that is compatible with our strategy of measuring the separability between classes. This goal can be accomplished as follows. Suppose that a given sequence *d_i _*has been correctly classified at taxonomic level *t*. Then, to classify it one level below in the taxonomic tree, *t *+ 1, we can eliminate all of the

sequences that do not belong to the same class as *d_i _*at level *t*. This procedure corresponds to selecting a specific classifier in the hierarchical scheme described above. Next, if we remove our initial assumption that *d_i _*was correctly classified at level *t*, it is clear that, by eliminating the appropriate sequences of the dataset, an incorrect classification at level *t *can be followed by a correct classification at level *t *+ 1. This strategy is precisely what allows us to evaluate the hierarchical classification using nothing but the nearest neighbor of each DNA sequence.

Observe that eliminating the sequences that do not belong to the same class as *d_i _*at level *t *corresponds to assuming that *d_i _*was correctly classified at that level. Of course, to have an accurate score function at level *t *+ 1, we must account for the possibility that the sequence was incorrectly classified at level *t*. Clearly, a straightforward way to estimate the probability of a misclassification at level *t *is to use the score function at that level. Therefore, we define the *hierarchical score *measure recursively: roughly speaking, the hierarchical score at level *t *corresponds to the product between the conventional score at the same level and the hierarchical score one level above. Algorithm 2 provides a step-by-step description of how to compute the proposed hierarchical score measure.

**Algorithm 2**: hierarchical_score(*d*, *t*, *n*, *s*)

/* Computes the hierarchical score for a given set of DNA sequences */

**Input**: *d *∈ *D*, *t *∈ *T*, *n *∈ *N*, *s *∈ *S*

**Output**: Hierarchical score

1** if ***t *= 1 **then return **conventional_score(*d*, *t*, *n*, *s*) ;/* *i.e*., if *t *is "domain" */

2**else**

3score ← 0

4*m *← 0

5**foreach ***sequence d_i _*∈ *d ***do**

6*d*' ← *d *with only sequences *d_k _*which belong to the same class as *d_i _*at level *t *- 1

7**if **|*d*'| > 1 *and d_i _is not the only representative of its class at level t ***then**

8*m *← *m *+ 1

9*d_j _*← NN(*d_i_*, *d*', *n*, *s*)

10**if **class(*d_i_*) = class(*d_j_*) at taxonomic level *t ***then **score ← score + 1

11**return **score/m * hierarchical_score(*d*, *t *- 1, *n*, *s*)

Using Algorithm 2, one can assess the degree of separability of taxonomic classes under a hierarchical classification scheme without making any strong assumptions regarding the shape of the classes. Therefore, the result of such an analysis applies to any set of classifiers, including a heterogeneous hierarchy composed of classifiers of different types.

## Results and discussion

As described above, in this work we assume that a given configuration of the taxonomic classification problem is defined by: (i) *n*, the length of *n*-mers used to encode the sequences; (ii) *s*, the similarity measure used; and (iii) *a*, the score measure, which can be the conventional measure or the hierarchical measure (Algorithms 1 and 2, respectively). To provide an empirical basis for the development of composition-based metagenomic classifiers, we analyzed the separability of taxonomic classes under different configurations of the classification task.

We performed 10 * 4 * 2 = 80 experiments with the genomic dataset and 8 * 4 * 2 = 64 experiments with the synthetic metagenomic fragments data (in both cases the three numbers correspond to *|N|*, *|S|*, and *|A|*, respectively; see Equation (5)). In total, we performed 80 + 64 = 144 experiments. Our analysis addresses the impact of parameters *n*, *s*, and *a *over the configuration scores. Although we also discuss other taxonomic levels, we focus our analysis on the classification problem at the taxon species.

### Complete genomes

The genomic dataset comprises 535 genomes encompassing 386 different species. Considering the conventional score measure, the configuration scores for this type of data at the level of species varied from *f *(*G*, *sp*, 1, *kl*, *c*) = 0.275, for the worst configuration, to *f *(*G*, *sp*, 5, 2, *c*) = 0.512, for the best configuration. The hierarchical scores varied between *f *(*G*, *sp*, 1, 2, *h*) = 0.378 and *f *(*G*, *sp*, 7, *kl*, *h*) = 0.532.

**Figure 2:**
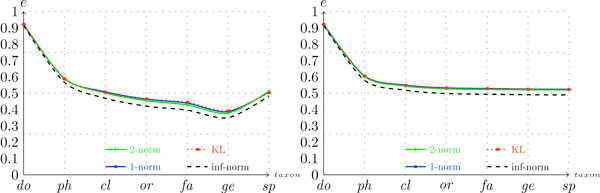
**Configuration scores per taxon for a genomic dataset (*d *= *G*)**. The graph on the left presents the scores for the configuration (*G*,*-*, 5, *-*, *c*) and graph on the right presents the scores for the configuration (*G*, *-*, 5, *-*, *h*).

Figure 2 presents the configuration scores that were generated on the genomic dataset over the different taxa for *n *= 5 (this value was the value of *n *that generated the highest conventional scores). As shown in the figure, as we go downward in the taxonomic tree (t → species), the configuration score decreases. This decrease is expected, because a correct nearest-neighbor classification at level *t *implies a correct classification at level *t - *1 (but not the converse). Observe that in the left graph in Figure 2 the score function actually increases when one moves from the taxon genus to the species. This increase is due to the removal of unique representatives of some species, as explained above. Surprisingly, varying the similarity measure *s *did not result in remarkable differences in the scores. As shown in Figure 2, the scores referring to *s *= 1, *s *= 2, and *s *= *kl *are very similar, and the scores computed with *s *= ∞ differ only slightly from the others. This phenomenon was observed across all configurations. Thus, from this point on, we will fix the similarity measure at *s *= *kl *and study the impact of the other variables over the scores.

**Figure 3:**
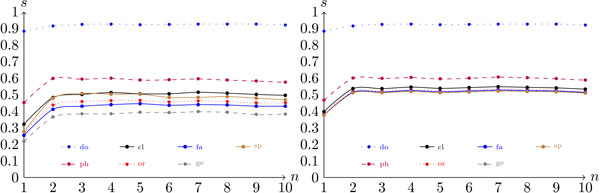
**Configuration scores per ***n***-mer word length for the genomic dataset**. The graph on the left was generated under the configuration (*G*, *-*,*-*, *kl*, *c*), and graph on the right was generated under (*G*, *-*,*-*, *kl*, *h*). All of the seven taxonomic levels are considered.

**Figure 4:**
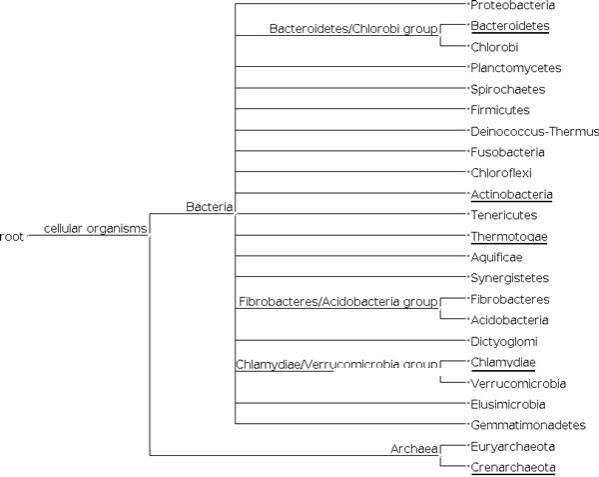
**Taxonomic tree**. Taxonomic tree for phyla, including Crenarchaeota, Actinobacteria, Bacteroidetes, Thermotogae, and Chlamydiae.

Figure 3 shows the genomic scores per *n*-mer length for the different taxa. In Figure 3, it is difficult to identify the value of *n *that produces the best score, because from *n *= 2 to *n *= 8 the score curve is almost flat. From *n *= 1 to *n *= 2 there is a rough increase in the scores. This increase was expected, because *n *= 1 means counting the frequencies of the nucleotides A, T, C, and G, which does not provide sufficient information about the sequences to discriminate between the classes. In general, a small value for *n *represents two different sequences in a similar way. As an example, consider the taxonomic tree shown in Figure 4, which includes the phyla Crenarchaeota, Actinobacteria, Bacteroidetes, Thermotogae, and Chlamydiae. Although these phyla are distant from a taxonomic point of view, some of their members give rise to very similar frequency vectors, as shown in Table 1.

**Table 1:** 1**-**mer frequencies for sequences in five different phyla.

Sequence	Sequence representation				Phylum
*d*_1_	0.2613	0.2611	0.2379	0.2397	Bacteroidetes
*d*_2_	0.2606	0.2612	0.2390	0.2392	Actinobacteria
*d*_3_	0.2445	0.2443	0.2557	0.2554	Thermotogae
*d*_4_	0.2430	0.2584	0.2439	0.2547	Chlamydiae
*d*_5_	0.2690	0.2678	0.2317	0.2315	Crenarchaeota

Observe also that from *n *= 8 to *n *= 10 the scores decrease slightly. This decrease is a consequence of the fact that, when *n *≥ 8, the number of possible *n*-mer sequences is very large, which results in sparse frequency vectors with a low discriminative power. For example, if the similar sequences *di *= *A***A***ATGGTA *and *d_j _*= *A***G***ATGGTA *are encoded with *n *= 8, the result is two vectors with 65, 536 positions filled with zeros in all but one position, which would contain a "1" representing the words *d_i _*and *d_j_*. Hence, we have two extremely similar sequences represented by two different frequency vectors, which clearly disrupts the score function *f*.

Concerning the two score measures, the hierarchical approach presented slightly better performance than the conventional score, as shown in Figures 2 and 3. This relationship suggests that decomposing the classification task into smaller sub-problems does indeed make the problem easier.

### Synthetic metagenomic fragments

The synthetic dataset comprises 23, 000 fragments with approximately 400*bp*. As mentioned previously, these sequences were generated with the sequences simulator MetaSim [16] under the sequencing conditions of the 454 pyrosequencer. The configuration scores at the level of species varied between *f*(*F*, *sp*, 8, ∞, *c*) = 0.007 and *f*(*F*, *sp*, 4, *kl*, *c*) = 0.112 for the conventional score function, and between *f*(*F*, *sp*, 7, 1, *h*) = 0.113 and *f*(*F*, *sp*, 4, 1, *h*) = 0.5 when the hierarchical measure was considered.

**Figure 5:**
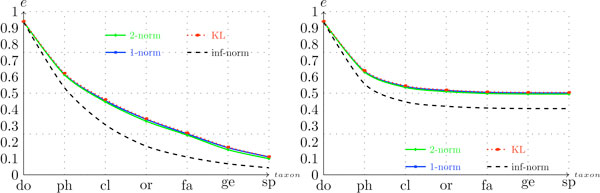
**Configuration scores per taxon for the metagenomic synthetic fragments dataset **(*d *= *F*)****. The graph on the left was generated under configuration (*F*, *-*, 4, *-*, *c*) and the graph on the right was generated under configuration (*F*, *-*, 4, *-*, *h*).

Figure 5 shows the value of the score as a function of the taxonomic level *t *when *n *= 4. The first thing that stands out in this figure is the fact that, for the synthetic data, the advantage of using the hierarchical score measure over the conventional measure is much more expressive than with complete genomes. This result indicates that, when sequences are short, the overlap between the classes is less correlated with the taxonomic tree. In other words, the overlap between two classes at level *t *is not strongly affected by the fact that they belong to the same class at level *t *- 1. A possible explanation for this phenomenon is that shorter sequences give rise to higher variability within each class.

**Figure 6:**
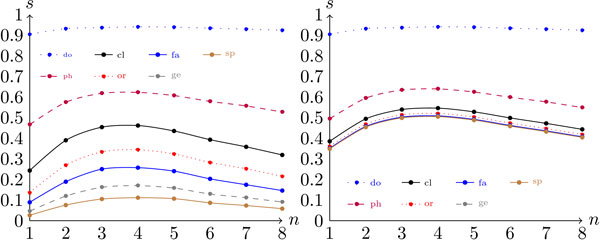
**Configuration scores per ***n***-mer word length for the metagenomic dataset**. The graph on the left was generated under the configuration (*F*, *-*,*-*, *kl*, *c*) and graph on the right was generated under the configuration (*F*, *-*,*-*, *kl*, *h*). All of the seven taxonomic levels are considered.

Again, changing the similarity measure *s *did not have a significant impact on the scores. Note, however, that with metagenomic fragments the use of *s *= ∞ has a degenerating impact over the scores which is more noticeable than the trend observed in the case of complete genomes (compare Figures 2 and 5). Figure 6 shows the conventional and hierarchical scores as a function of *n *when the KL divergence is adopted as the similarity measure. Here we observe curves similar to the curves shown in Figure 3, with the peak of each curve shifted slightly to the left. This change makes sense, because with shorter sequences the "sparsification" of frequency vectors discussed in the previous section occurs at smaller values of *n*. Additionally, note how the conventional scores of the metagenomic dataset are low at the taxonomic level of order and below. This trend suggests that using a single classifier in this case might not be the best alternative. Such an observation could be particularly helpful in the future development of composition-based classifiers, because one of the major problems with real metagenomic projects is the difficulty of obtaining accurate classification at lower taxonomic levels [8,12].

In summary, we observed that the scores associated with metagenomic data are in general smaller than the scores generated with genomic data, and using a hierarchical classification approach in this case appears to be even more beneficial. Moreover, the value of *n *that generated the best results decreased from *n ≈ *7 to *n *≈ 4, which indicates that, when dealing with metagenomic fragments with approximately 400*bp*, there is no point in using frequency vectors that have a dimension much higher than 256.

### Discussion

**Figure 7:**
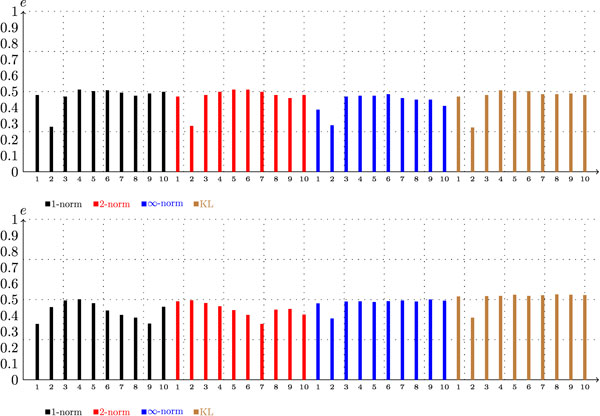
**Scores as a function of *n *and *s *for a genomic dataset (*d *= *G*)**. The *x*-axis represents the length of the *n*-mer sequences. The top graph is the conventional score function (*G*, *sp*, *-*,*-*, *c*), and the bottom graph is the hierarchical score function (*G*, *sp*, *-*,*-*, *h*).

**Figure 8:**
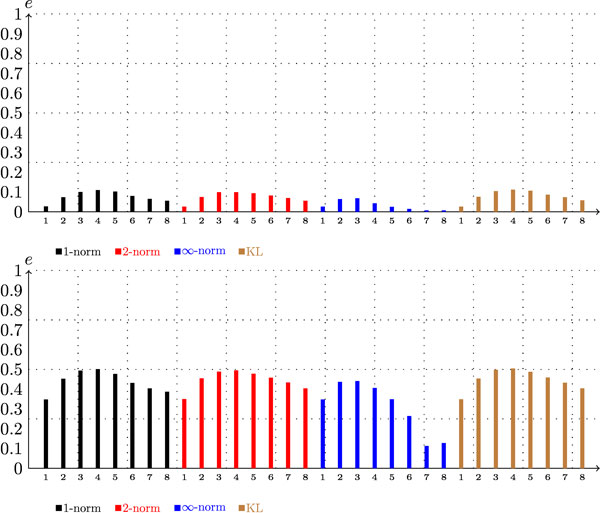
**Scores as a function of *n *and *s *for synthetic metagenomic dataset **(*d *= *F*)****. The *x*-axis represents the length of the *n*-mer sequences. The top graph is the conventional score function (*F*, *sp*, *-*,*-*, *c*), and the bottom graph is the hierarchical score function (*F*, *sp*, *-*,*-*, *h*).

In this section we summarize the results presented in the previous sections and provide an overview of our analysis. To accomplish those goals, we show in Figure 7 the scores that were generated with the genomic data at the level of species as a function of *n *and *s*, and in Figure 8 we show the same information for the scores generated with the metagenomic dataset. From examining these figures, we arrive at the following conclusions:

*• *The scores are an approximately concave function of *n *with a maximum value that is between 4 and 7; the "optimal" value of *n *is smaller for the metagenomic dataset.

*• *Changing the similarity measure *s *does not have a strong effect on the scores.

*• *The hierarchical classification scheme appears to be a better alternative for both genomic and metagenomic data; however, in the latter case, its advantage over the conventional classification approach is more evident.

*• *In general the scores that are associated with the metagenomic data are smaller than the scores that are associated with the genomic data, but the difference is more significant under the conventional classification scheme.

In conclusion, we show in Table 2 the configurations that produced the best results in both datasets. The values shown in Table 2 can serve as a starting point in the development of composition-based metagenomic classifiers.

**Table 2:** Configuration scores referring to the best *n *and *s *values.

	Conventional			Hierarchical		
	*n*	*s*	*score*	*n*	*s*	*score*
Genomes	5	2	0.512	7	kl	0.532
Fragments	4	kl	0.112	4	1	0.501

The scores were produced considering both score measures and both datasets, at the species taxonomic level.

## Conclusions

Taxonomic classification is an essential step within a metagenomic study, since this is the first step of a metagenomic analysis and its result is used as a basis to posterior investigations. Usually, composition-based metagenomic classifiers are configured based on preliminary experiments that account for a specific type of classifier. In this work we proposed to shift the focus of the analysis to the classification task itself. To make this shift, we presented a general framework that can be used to study the impact of several decisions on the difficulty of the classification problem (that is, how "separable" the classes are under different configurations of the task).

In this work we focused the analysis on the impact of three factors in particular: (i) the length of the *n*-mers used to encode the DNA sequences; (ii) the similarity measure used to compare frequency vectors; and (iii) the underlying classification scheme (hierarchical or conventional). The results presented provide some intuition on how the difficulty of the classification problem changes as a function of the features above. Because our analysis does not assume any structure of the classification problem, it can be used as a guideline for the development of composition-based metagenomic classifiers of any type. Moreover, the framework presented in this work can be used for the analysis of the impact of other factors over the taxonomic classification task.

## Competing interests

The authors declare that they have no competing interests.

## Authors' contributions

SH performed the experiments, helped in the interpretation of the results, and wrote the manuscript. AMSB analyzed and interpreted the results, and helped in writing the manuscript. MEC helped in the experiments, and reviewed the manuscript. ATRV reviewed the manuscript, and conceived the project. All of the authors have approved the final version to be published.
